# Genetic Mechanism for the Cyclostome Cerebellar Neurons Reveals Early Evolution of the Vertebrate Cerebellum

**DOI:** 10.3389/fcell.2021.700860

**Published:** 2021-08-18

**Authors:** Fumiaki Sugahara, Juan Pascual-Anaya, Shigehiro Kuraku, Shigeru Kuratani, Yasunori Murakami

**Affiliations:** ^1^Division of Biology, Hyogo College of Medicine, Nishinomiya, Japan; ^2^Evolutionary Morphology Laboratory, RIKEN Cluster for Pioneering Research (CPR), Kobe, Japan; ^3^Department of Animal Biology, Faculty of Sciences, University of Málaga, Málaga, Spain; ^4^Andalusian Centre for Nanomedicine and Biotechnology (BIONAND), Málaga, Spain; ^5^Laboratory for Phyloinformatics, RIKEN Center for Biosystems Dynamics Research (BDR), Kobe, Japan; ^6^Molecular Life History Laboratory, Department of Genomics and Evolutionary Biology, National Institute of Genetics, Mishima, Japan; ^7^Laboratory for Evolutionary Morphology, RIKEN Center for Biosystems Dynamics Research (BDR), Kobe, Japan; ^8^Graduate School of Science and Engineering, Ehime University, Matsuyama, Japan

**Keywords:** cerebellum evolution, cyclostome, lamprey, hagfish, evolutionary developmental biology (EvoDevo), rhombic lip, Purkinje cells, granule cells of cerebellum

## Abstract

The vertebrate cerebellum arises at the dorsal part of rhombomere 1, induced by signals from the isthmic organizer. Two major cerebellar neuronal subtypes, granule cells (excitatory) and Purkinje cells (inhibitory), are generated from the anterior rhombic lip and the ventricular zone, respectively. This regionalization and the way it develops are shared in all extant jawed vertebrates (gnathostomes). However, very little is known about early evolution of the cerebellum. The lamprey, an extant jawless vertebrate lineage or cyclostome, possesses an undifferentiated, plate-like cerebellum, whereas the hagfish, another cyclostome lineage, is thought to lack a cerebellum proper. In this study, we found that hagfish *Atoh1* and *Wnt1* genes are co-expressed in the rhombic lip, and *Ptf1a* is expressed ventrally to them, confirming the existence of r1’s rhombic lip and the ventricular zone in cyclostomes. In later stages, lamprey *Atoh1* is downregulated in the posterior r1, in which the *NeuroD* increases, similar to the differentiation process of cerebellar granule cells in gnathostomes. Also, a continuous *Atoh1*-positive domain in the rostral r1 is reminiscent of the primordium of valvula cerebelli of ray-finned fishes. Lastly, we detected a *GAD*-positive domain adjacent to the *Ptf1a*-positive ventricular zone in lampreys, suggesting that the *Ptf1a*-positive cells differentiate into some GABAergic inhibitory neurons such as Purkinje and other inhibitory neurons like in gnathostomes. Altogether, we conclude that the ancestral genetic programs for the formation of a distinct cerebellum were established in the last common ancestor of vertebrates.

## Introduction

**FIGURE 1:**
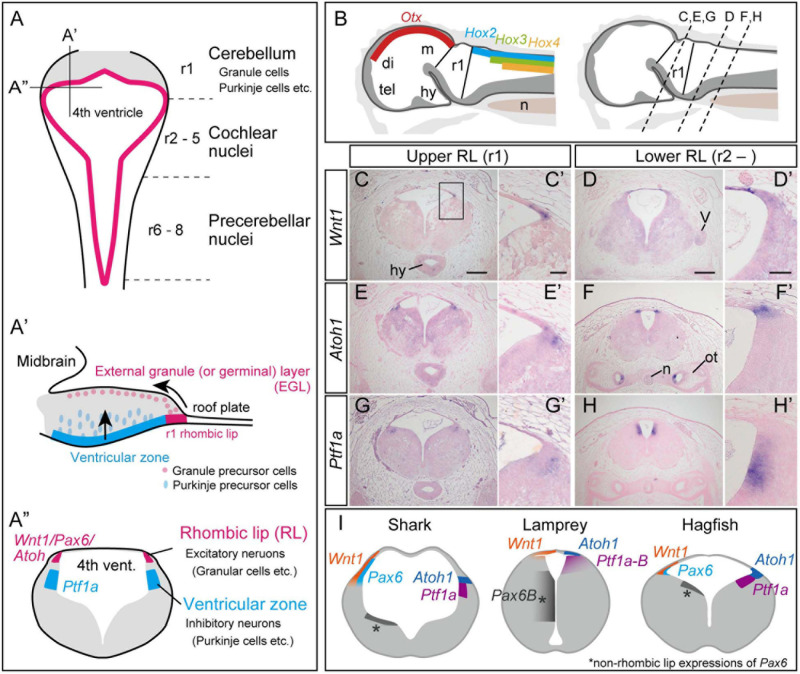
Development of the cerebellum in mammals, based on Fujiyama et al., 2009 **(A)**. **(A)** Dorsal view of the rhombomeres. The rhombomere 1 (r1) gives rise to the cerebellum, while r2–8 generates the cochlear nuclei and precerebellar nuclei. **(A’)** Sagittal view of the rhombomere 1. Granule precursor cells, generated in the r1 rhombic lip, migrate tangentially to form the external germinal layer (EGL). The Purkinje precursor cells originating from the ventricular zone secrete Shh, which is involved in the proliferation of the granule cells. The granule cells in EGL then migrate ventrally to form the granular layer. **(A”)** Transverse view of rhombomere 1 showing the rhombic lip as the source for excitatory neurons, and the ventricular zone where the inhibitory neurons arise. **(B–H)** Upper and lower rhombic lip gene expression patterns in the hagfish, *E. burgeri*. **(B)** Illustration of a sagittal section of the anterior part of a stage 53 embryo. (left) Rhombomere 1 (Upper rhombic lip) is identified as a negative region of both *Otx* and *Hox* genes (Oisi et al., 2013; Pascual-Anaya et al., 2018). (right) Anterior-posterior level of transverse sections shown in **(C–H)** are indicated in a sagittal illustration. **(C–H)** Section *in situ* hybridizations for hagfish *Wnt1*, *Atoh1* and *Ptf1a* genes showing their expression patterns in r1 **(C,E,G)** and posterior to r1 **(D,F,H)**. **(C’–H’)** Higher magnification images of the rhombic lip expression patterns shown in **(C–H)**. **(I)** Comparison of the upper rhombic lip gene expression patterns in the shark (*S. torazame*), lamprey (*L. camtschaticum*), and hagfish (*E. burgeri*) based on Sugahara et al. (2017) and the present study. di, diencephalon; hy, hypothalamus; m, mesencephalon; n, notochord; ot, otic capsule; tel, telencephalon; r1, rhombomere 1; V, trigeminal nerve. Scale bars: 200 μm **(C–H)**, 50 μm **(C’–H’)**.

During development, the cerebellum arises from the dorsal part of rhombomere 1 (r1), just posterior to the midbrain-hindbrain boundary (MHB or isthmic organizer). R1 is defined as an *Otx*- and *Hox*-negative region and this molecular state is critical for cerebellar development (Broccoli et al., 1999; Katahira et al., 2000; Butts et al., 2014a; Figure 1B). In addition, FGF8, a secreted molecular signal from the MHB, plays an important role for the induction of the cerebellum (Reifers et al., 1998; Sato et al., 2001). *Wnt1*, which is expressed in the most caudal part of the midbrain, is also known to encode an important signaling molecule. Although not directly involved in the induction of the cerebellum, *Wnt1*-knockout mice lack the cerebellum. This, however, might be a secondary effect as a result of the disruption of the specification of the midbrain and hindbrain (McMahon and Bradley, 1990).

Excitatory granule neurons (glutamatergic), and inhibitory Purkinje cells (GABAergic) play a major role in the processing of the cerebellar cortex. These cerebellar neurons arise from two distinct embryonic domains: the rhombic lip and the ventricular zone (Figures 1A–A”). The rhombic lip is the rostrodorsal edge of the hindbrain formed just below the roof plate (Wullimann et al., 2011). The rhombic lip can be divided into the anterior part that arises from r1 and the rest, the posterior part comprised between r2 and 8. In gnathostomes, *Wnt1* and *Pax6* are co-expressed throughout the rhombic lip. Also, *Atoh1* (a bHLH transcription factor; *Math1* in mice)-positive neuron precursors specifically arise from the upper (anterior) rhombic lip, differentiating into cerebellar granule cells, while the cells in the lower (posterior) rhombic lip differentiate into excitatory neurons in the cochlear nuclei and the precerebellar nuclei and give rise to mossy fibers (Fujiyama et al., 2009). In amniotes, *Atoh1*-positive precursors migrate secondarily from the upper rhombic lip to cover the surface of the cerebellar cortex, forming the external granule cell layer (EGL) (Wullimann et al., 2011; Figures 1A–A”). The proliferative activity of EGL cells is stimulated transiently by Shh signaling from Purkinje cells (Martí and Bovolenta, 2002). Subsequently, the EGL cells start to express a proneural gene, *NeuroD1*, and stop expressing *Atoh1*, then shifting toward a postmitotic state. Finally, the granule cells migrate ventrally to generate an internal granular layer (Wullimann et al., 2011). This proliferative EGL may have been acquired in the amniote lineage, because frogs have only non-proliferative EGL (Gona, 1972; Butts et al., 2014b) and no EGL has been identified in zebrafish, paddlefish (*Polyodon spathula*), and sharks (*Scylliorhinus canicula*) (Chaplin et al., 2010; Butts et al., 2014c; Hibi et al., 2017). However, there are reports of cell migration and genetic mechanisms similar to the amniote EGL in zebrafish (Biechl et al., 2016), although this remains controversial. The internal migration of the granule cells is also reported in frogs and teleosts, but not in sharks and paddlefish (Chaplin et al., 2010; Butts et al., 2014c; Hibi et al., 2017).

The ventricular zone, another source of cerebellar neurons, occupies the ventromedial position to the rhombic lip (Figure 1A”). This domain is characterized by the expression of the bHLH transcription factor coding gene *Ptf1a*, and gives rise to GABAergic inhibitory neurons including Purkinje, basket, stellate, and Golgi cells in the cerebellar cortex (Leto et al., 2016). A *Ptf1a* knockout mouse, *cerebelles*, lacks the entire cerebellar cortex together with Purkinje cells and other GABAergic interneurons, indicating that *Ptf1a* is essential for the development of GABAergic inhibitory neurons in the cerebellum (Hoshino et al., 2005; Hoshino, 2006). The caudal area (r2-r8) of the ventricular zone produces inhibitory neurons in the cochlear nuclei and neurons that generate climbing fibers in the precerebellar nuclei (Yamada et al., 2007; Fujiyama et al., 2009).

Cyclostomes that include lampreys and hagfish, are the only living jawless vertebrates and diverged from gnathostomes over 500 million years ago (Kuraku and Kuratani, 2006). The lamprey possesses only a small, plate-like cerebellum in the rostrodorsal part of the hindbrain, in which small granule-like cells are present (Striedter and Northcutt, 2020). Therefore, the lamprey cerebellum is considered to be homologous to the corpus cerebelli (the central lobe of the cerebellum) of gnathostomes (Nieuwenhuys, 1967). However, the lamprey lacks a layered cerebellar cortex. The presence of the Purkinje cells is still ambiguous in lamprey (Lannoo and Hawkes, 1997). On the other hand, the hagfish, the other group of cyclostomes, does not possess a morphologically distinct cerebellum, showing only the vestibulo-lateral commissure reported at the midbrain-hindbrain boundary (Larsell, 1947; Nieuwenhuys et al., 1998; Sugahara et al., 2016).

Previous studies have shown that lamprey embryos exhibit *Otx*- and *Hox1*-negative rhombomere 1 region (Ueki et al., 1998; Takio et al., 2004), while *Gbx* is expressed in the entire hindbrain (Takio et al., 2007). As in gnathostomes, the lamprey MHB expresses *FGF8* and *Wnt1* (Shigetani et al., 2002; Sugahara et al., 2016; Supplementary Figure 1), suggesting that a certain regionalization and inductive signals for the cerebellum are shared at least by lampreys. Regarding the development of cerebellar neurons, we previously reported that *Atoh1* is expressed in the lamprey in dorsal rhombomeres, including r1, and one of two orthologous genes of *Ptf1a* expressed in the ventral part (Sugahara et al., 2016). However, it is still unclear whether cerebellar neuron subtypes are differentiated from these domains or not. Much less is known about the presence or absence of this regionalization in the hagfish, the involved induction signals or the genetic program for the specification of cerebellar neurons subtypes. We previously reported that *Pax6* and *Atoh1* are expressed in the rhombic lip of the hagfish but could not identify exactly if it was in r1 only or also in more caudal rhombomeres, because we could use only sectioned samples due to the limitation of embryonic material (Ota et al., 2007). Here, we further investigated the molecular basis underlying the cerebellum regionalization and the cerebellar neuron specification in lamprey and hagfish embryos. Also, by comparing gnathostomes and cyclostomes, we tried to depict the molecular and morphological ground patterns of the cerebellum of the last common ancestor of crown vertebrates, and to infer the evolutionary changes in its developmental mechanisms in the different vertebrate lineages.

## Materials and Methods

### Animals

Matured arctic lamprey, *Lethenteron camtschaticum* were collected in Hokkaido, Japan. Embryos were obtained by artificial fertilization and staged as described previously (Tahara, 1988; Sugahara et al., 2015) and fixed with 4% paraformaldehyde. Matured Japanese inshore hagfish, *Eptatretus burgeri* were caught from the Japan Sea off Shimane Prefecture. The embryos of *E. burgeri* were obtained by keeping adults in cages and placed in the sea bottom as described previously (Oisi et al., 2015). The staging of hagfish embryos followed Dean (1899) and Oisi et al. (2013) and fixed with Serra’s fixative (6:3:1 mixture of ethanol, formalin, and glacial acetic acid).

### Gene Identification and Phylogenetic Analysis

Sequence of hagfish *Wnt1* and *Ptf1a* genes were obtained from a previously published transcriptome assembly (Pascual-Anaya et al., 2018) by means of TBLASTN v2.2.31 + searches (Altschul et al., 1997) and using gnathostome counterparts as queries. *Ptf1a* sequence was completed on the 3′ end by manual cloning and sequence following standard protocols. For the lamprey *GAD* gene, the sequence was obtained from transcriptome data reported previously (Pascual-Anaya et al., 2018), based on the sequence of *Petromyzon marinus GAD1* (XM_032965485). Reverse transcription polymerase chain reaction (RT–PCR) was performed to amplify fragments of each gene with specific primers from total RNA of lamprey embryos or hagfish juvenile (sequences of primers used are listed in Supplementary Table 1). The sequences identified here have been deposited in GenBank under accession numbers LC60429-31. Hagfish *Atoh1* (KT897935), lamprey *Atoh1* (KT897930), *Ptf1a-B* (KT897934), *NeuroD* (LC424505) were reported previously (Sugahara et al., 2016; Higuchi et al., 2019).

Molecular phylogenetic tree inference of the *Wnt1* and *Ptf1a* genes were performed as previously described (Hara et al., 2018) using homolog sequences retrieved via the aLeaves webserver (Kuraku et al., 2013).

### *In situ* Hybridization of the Hagfish and Lamprey Embryos

*In situ* hybridization on paraffin sections of *E. burgeri* was performed as described (Oisi et al., 2015). Whole-mount *in situ* hybridization on lamprey embryos was performed according to Sugahara et al., 2015. Some stained specimens were made transparent by 1:2 mixture of Benzyl-alcohol and Benzyl-benzoate after dehydration. Staining specimens were embedded in tissue tek^®^ O.C.T.^TM^ compound and sectioned (12 μm) by CryoStar NX50 (Thermo Fisher Scientific).

## Results

### Isthmic Gene Expressions in Cyclostomes

Previously, we have identified the expression of *FGF8/17* and *Wnt1* in a sharp domain, of the lamprey brain and corresponding to the MHB (Shigetani et al., 2002; Sugahara et al., 2016; Supplementary Figures 1B,D). However, the presence of this expression domain in the hagfish is so far unknown. We newly identified a hagfish *Wnt1* orthologous gene (Supplementary Figure 3A) and investigated its expression pattern in the hagfish brain. At mid-pharyngula stage (Dean stage 45), hagfish *Wnt1* expression was observed as a sharp transverse band corresponding to the MHB (Supplementary Figure 1A). Also, hagfish *FGF8/17* was expressed around the MHB, but in a relatively broader domain than that of lamprey and gnathostomes (Supplementary Figure 1C).

### Expression Patterns of Upper- and Lower-Rhombic Lip Marker Genes in Hagfish

Next, we investigated whether the genetic machinery for r1-neuronal differentiation is present in cyclostomes or not. Generally, when looked from a dorsal view, the gnathostome hindbrain appears as a rhomboid shape with a thin roof plate over the fourth ventricle. The most expanded region corresponds to the boundary between r1 and r2 (Wingate, 2001). In hagfish, the anterior border of the *Hox2* expression was seen in the lateral edge of the fourth ventricle (Pascual-Anaya et al., 2018). We therefore defined this region as the posterior limit of the anterior rhombic lip (Figure 1B). At late pharyngular stage (Dean stage 53), *Atoh1* was expressed in the bilateral dorsal-most edges of the hindbrain, the “rhombic lip,” in the upper and lower rhombomeres. *Wnt1* expression overlaps with *Atoh1* in the rhombic lip but extends to the roof plate (Figures 1C–F’). We newly identified a *Ptf1a* ortholog gene in hagfish (Supplementary Figure 3B). Hagfish *Ptf1a* is expressed at the surface of the fourth ventricle ventral to the *Atoh1/Wnt1* expressing region in both lower and upper rhombomeres (Figures 1G,H). These results indicate that the regionalization of the rhombic lip and the ventricular zone are present in hagfish r1 (Figure 1I).

### Spatio-Temporal Atoh1 Expression in the Lamprey Hindbrain

**FIGURE 2:**
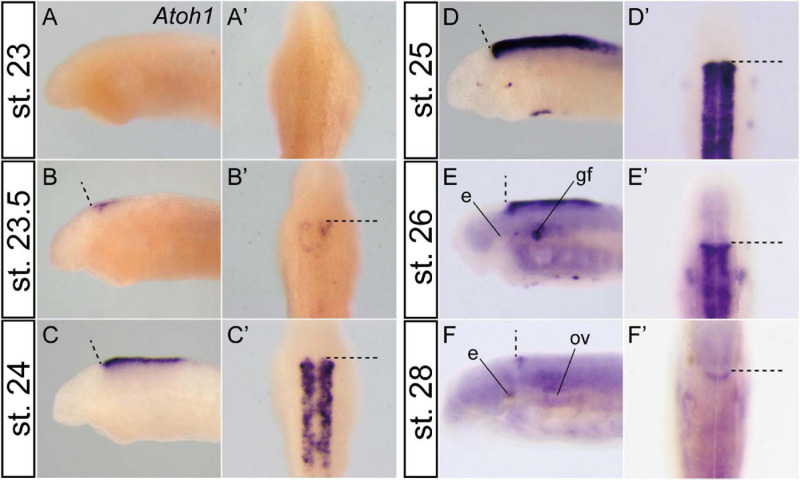
Temporal *Atoh1* expression pattern in the lamprey hindbrain. Lateral **(A–F)** and dorsal views **(A’–F’)**. Dashed lines indicate the MHB. *Atoh1* expression starts at stage 23.5 in the rostral half of the rhombic lip **(B)** and subsequently expands throughout the rhombic lip during stages 24–26 **(C–E)**. After stage 27 (not shown), the expression is restricted only to the rostral border of the rhombomere 1 **(F)**. Note that the apparent asymmetrical staining in **(B)** does not reflect the actual difference in expression, but is caused by the staining process. e, eye; gf, complex of facial/lateral line ganglion; ov, otic vesicle.

In zebrafish and paddlefish, granule neuron precursors are reported to express *Atoh1* in early embryonic stages, but *Atoh1* is later downregulated in the lateral cerebellar rhombic lip. Subsequently, post-mitotic granule precursor cells express *NeuroD* and differentiate into matured granule cells. Meanwhile, proliferation of the precursor cells, which express *Atoh1*, is selectively maintained in the rostromedial rhombic lip until late development (Kani et al., 2010; Butts et al., 2014c). To investigate whether the same or a similar genetic mechanism for the differentiation of the cerebellar granule cells is present in the lamprey or not, we observed the spatio-temporal expression pattern of *Atoh1* in lamprey embryos. *Atoh1* expression starts at stage 23.5 in the rostral half of the rhombic lip (Figure 2B) and then expands throughout the upper and lower rhombic lip during stages 24–26 (Figures 2C–E). Subsequently, this *Atoh1* expression is downregulated in the caudal half of r1 and lower rhombic lip, whereas its expression is maintained in the rostral tip of r1. This continuous expression of *Atoh1* is observed at least until respiration stage (stage 28) (Figure 2F). Like ray-finned fish, the rostral area of lamprey r1 is apparently regulated differently from the other rhombic lip (Kani et al., 2010; Butts et al., 2014c). In birds and mammals, *Atoh1*-positive granule precursor cells migrate from the rhombic lip to cover the surface of the cerebellum to form the EGL. Subsequently, EGL cells migrate ventrally and form the internal granule layer (Butts et al., 2014a). In the lamprey, however, we could not observe the expansion or migration of *Atoh1*-positive cells as is the case in the shark and paddlefish (Chaplin et al., 2010; Butts et al., 2014c).

### Differentiation of Atoh1–Positive Cells in the Lamprey Late Development

**FIGURE 3:**
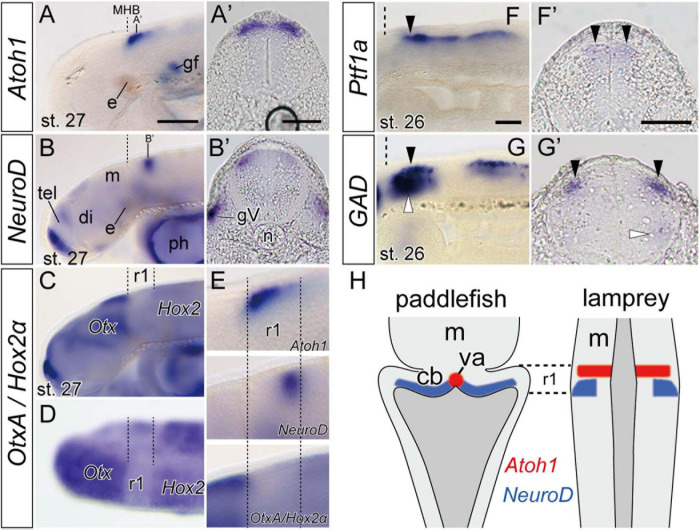
Expression of genes involved in neuron differentiation during lamprey late embryonic stages. **(A,A’)** *Atoh1* expression is restricted to the anterior r1 in stage 27 embryo (arrow heads). Dashed lines indicate the MHB. **(B,B’)** *NeuroD* expression is observed behind the *Atoh1* expression domain (arrowheads). Note that **(A’,B’)** are not at the same rostrocaudal positions in r1. The apparent asymmetrical staining in **(B’)** does not reflect the actual difference in expression, but is caused by the slightly oblique cutting of the sections. **(C,D)** *In situ* hybridization of both *OtxA* and *Hox2*α probes in stage 27 in lateral view **(C)** and dorsal view **(D)**. *OtxA* is expressed only anterior to the midbrain, whereas *Hox2*α is expressed caudally from r2. Dashed lines indicate the borders of r1 identified as “Otx–Hox free” domain. **(E)** High magnifications of the panel **(A–C)** in r1 region. **(F,F’)** *Ptf1a-B* expression at the ventricular zone at stage 26 (arrowheads). **(G,G’)** *GAD* is expressed in the hindbrain at the same dorsal level than –but lateral to– the *Ptf1a* expression domains (black arrowheads) and also in a ventral domain (white arrowheads). **(H)** Comparison of *Atoh1* and *NeuroD* expression patterns in the hindbrain between paddlefish, *Polyodon spathula* (stage 42, based on Butts et al., 2014c), and lamprey. Note that although the lamprey hindbrain does not show a ‘diamond shape’ of the rhombic lip during embryonic stages, the topological relationship of these gene expression patterns is comparable in both species. cb, cerebellum primordium; gV, trigeminal ganglion; ph, pharynx; va, valvula cerebelli. See Figures 1,2 for other abbreviations. Scale bars, 100 μm **(A–D)**, 50 μm **(A’,B’,F,F’,G,G’)**.

As mentioned above, lamprey *Atoh1* is downregulated at late stages except in the rostral area of the r1 (Figure 2F). In accordance with the decrease of *Atoh1* expression, the *NeuroD* expression become apparent slightly caudal to the *Atoh1*-positive area (Figures 3A,B and Supplementary Figure 2). To distinguish whether the *NeuroD*-positive region is included within r1 or not, we conducted double *in situ* hybridization with *OtxA* and *Hox2*α (Figures 3C,D). Since *OtxA* and *Hox2*α are expressed in the midbrain and r2, respectively (Ueki et al., 1998; Takio et al., 2004), we could identify r1 as the *OtxA*/*Hox2*α-negative region in between. By comparing *NeuroD* expression with *Otx–Hox2* expression, we identified the *NeuroD*–positive region located in the posterior part of r1 (Figure 3E). Transverse sections showed that *NeuroD* expression was located lateral to, but slightly overlapping the *Atoh1* expression domain (Figures 3A’,B’). The topological location of these two *Atoh1* and *NeuroD* expression domains suggests that precursors migrate from more anterior/medial (*Atoh1-*positive) areas to more posterior/lateral (*NeuroD-*positive) zones during their differentiation process. At the same time, however, neuron precursors in the rostral r1 retain *Atoh1* expression. This positional relationship is comparable to the development of cerebellar granule neurons in some gnathostome fishes as discussed below.

### *Ptf1a* Expression and GAD–Positive Inhibitory Neurons

In mammals, *Ptf1a*–positive cells in r1 ventricular zone differentiate into GABA-releasing inhibitory neurons such as Purkinje cells and several other types of interneurons (basket, stellate, and Golgi cells) (Hoshino et al., 2005). Presence of *Ptf1a*–positive cells in r1 ventricular zone in both lampreys and hagfish suggests that an active and probably homologous genetic mechanism for GABAergic inhibitory neuron production exist in cyclostomes. We therefore observed the expression of *glutamate decarboxylase* (*GAD*) gene, which encodes an enzyme to catalyze the synthesis of GABA. In lamprey, at stage 26, *GAD* expression domain overlaps the *Ptf1a* expression domain in the anterior part of the rhombencephalon (including r1) (arrowheads in Figures 3F,G). Transverse sections showed that *GAD* expression localizes relatively lateral to the *Ptf1a*–positive ventricular zone (Figures 3F’,G’). Since *Ptf1a* is expressed only in undifferentiated neurons in the neuroepithelium (Hoshino et al., 2005), this may possibly reflect that *Ptf1a*-positive precursor cells in lamprey are generated in the ventricular zone, then migrate radially, and finally differentiate into inhibitory GABAergic neurons.

## Discussion

### Presence of MHB Signals in Cyclostomes

Signaling factors from the MHB are essential for the induction and development of the gnathostome cerebellum (Leto et al., 2016). In this study, we identified both *FGF8/17* and *Wnt1* expression around the MHB region in the hagfish embryo similar to gnathostomes and the lamprey (Supplementary Figure 1), suggesting that these signals are conserved through all extant vertebrates. Curiously, hagfish *FGF8/17* expression showed a broader pattern than in other vertebrates (Supplementary Figure 1C). This unique *FGF8/17* expression pattern in the hagfish might be related to the absence of a distinct cerebellum in adult hagfish. Alternatively, FGF8 signaling might have a limited effect on cerebellar development, since some reports have indicated that conditional mutant mice with reduced *FGF8* expression in the MHB shows only loss of the cerebellar vermis region (Basson et al., 2008; Yu et al., 2011, 2013). Further experiments would be needed to explain this unusual *FGF8/17* expression pattern in the hagfish.

### Upper and Lower Rhombic Lip and Ventricular Zone in Cyclostomes

We morphologically defined the domain of r1 in paraffin sections of a hagfish embryo and found that *Wnt1* and *Atoh1* are expressed not only in r1, but also in the more caudal rhombomeres. Expression of *Pax6*, another rhombic lip gene, was previously reported (see Figure 4C in Sugahara et al., 2016). In addition, we identified *Ptf1a* expression in the ventral side of the *Wnt1/Atoh1* expression domain (Figures 1G,H). Together with previous studies in the lamprey, we conclude that the regionalization into the upper/lower rhombic lip and the accompanying ventricular zone is shared between cyclostomes and gnathostomes (Figure 1I).

### Presence of a Differentiation Mechanism for Granule Cells and a Continuous *Atoh1*-Expression Domain

Lamprey *Atoh1* is initially expressed in the entire rhombic lip, but subsequently gets downregulated in the caudal half of r1 and the lower rhombic lip (Figure 2). Concomitantly with this downregulation, *NeuroD* becomes upregulated in the caudal part of r1 (Supplementary Figure 2 and Figure 3B). This transition is consistent with the differentiation process of granule cells in zebrafish, paddlefish and amniotes (Kani et al., 2010; Butts et al., 2014c). Therefore, the genetic mechanism for the differentiation of the cerebellar granule cells might be present in the lamprey as well.

We found a continuous expression domain of the *Atoh1* in the rostral r1, topologically comparable to the rostromedial domain of the paddlefish and zebrafish r1 (Kani et al., 2010; Butts et al., 2014c; Figure 3H). In the zebrafish, this region differentiates into valvula cerebelli, a unique cerebellar structure in ray-finned fishes but not in cartilaginous fish and lobe-finned fish including tetrapods (Nieuwenhuys et al., 1998). Although the valvula cerebelli has not been identified anatomically in adult lampreys, it is possible that a homologous genetic domain might be present in the embryonic lamprey r1.

### Presence of GAD-Positive Inhibitory Neurons in Lamprey r1

In mice, all cerebellar inhibitory neurons arise from the *Ptfa1*-positive ventricular zone (Hoshino et al., 2005). Also, inhibitory neurons in the cochlear nucleus arise from r2–5, and the climbing fibers originate from the inferior olivary nucleus arise from r6–8 (Yamada et al., 2007; Fujiyama et al., 2009). In this study, orthologous *Ptf1a* genes were expressed in the ventricular zone in lamprey and hagfish r1 and more posterior rhombomeres (Figures 1, 3). In addition, lamprey *GAD and Ptf1a* expression domains were observed at the same anterior-posterior level in the rhombomeres with a distinct dorso-ventral pattern (Figure 3G). If *GAD*-positive inhibitory neurons are derived from the *Ptf1a*-positive ventricular zone, GABAergic inhibitory neurons in the lamprey may be formed by a similar developmental mechanism to that of gnathostome Purkinje cells. However, so far, Purkinje cells have not been identified in the adult lamprey cerebellum (Nieuwenhuys et al., 1998; Striedter and Northcutt, 2020). Further studies are necessary to determine whether these GABAergic neuron precursors in the lamprey embryo are homologous to Purkinje cells or any other inhibitory interneurons in gnathostomes or not (e.g., basket, stellate, and Golgi cells). In mice, small GABAergic neurons in the deep cerebellar nuclei (DCN) arise from the *Ptf1a*-positive ventricular zone and migrate ventrally (Hoshino et al., 2005). It is necessary to confirm whether the ventral population of *GAD*-expressing cells in the lamprey (white arrowheads in Figure 3G) originate from the ventricular zone and migrate ventrally like DCN neurons in amniotes. In mice, a subpopulation of *Ptf1a-*positive cells in the ventricular zone expressing *Gsx1* differentiates into interneurons in the DCN (Seto et al., 2014). Two *Gsx1* homolog genes have been identified in the lamprey and one of them was amplified from the brain tissue by RT-PCR (Zhang et al., 2017). However, further research is needed to determine whether a similar neurodevelopmental mechanism for the differentiation of *Ptf1a-*expressing cells in the ventricular zone is present in the lamprey.

### Developmental Evolution in the Vertebrate Cerebellum

**FIGURE 4:**
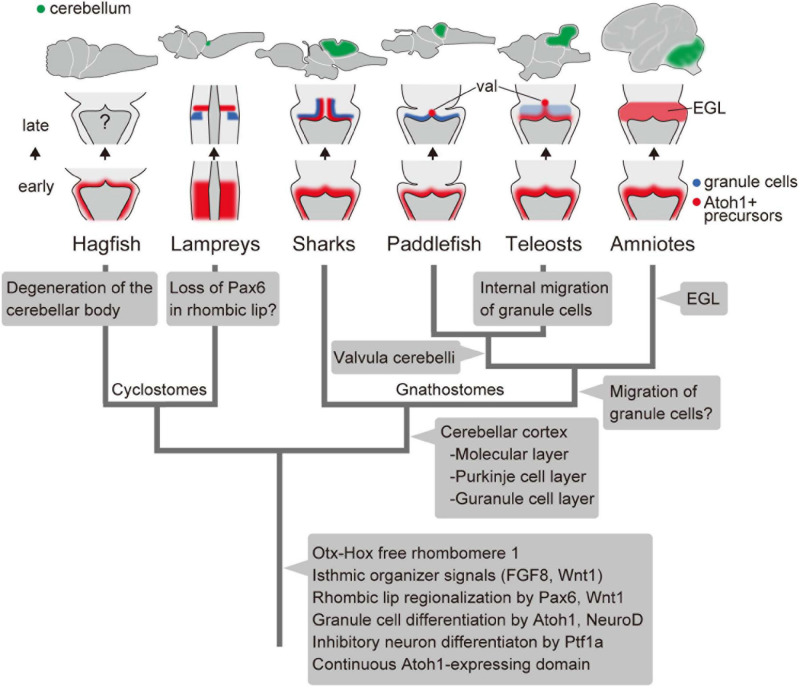
An evolutionary scenario for the vertebrate cerebellum, based on Chaplin et al. (2010) and Butts et al. (2014c) and the present study. Note that the internal migration of postmitotic granule cells has been observed in teleosts but not in the paddlefish. Therefore, it is equally possible that this migration of granule cells was acquired after the divergence of chondrichthyans and osteichthyans, with paddlefish losing this ability secondarily. Alternatively, it is possible that teleosts acquired the migration independently from amniotes. val, valvula cerebelli; EGL, external granule cell layer.

By comparing both cyclostomes and gnathostomes, we can depict a hypothetical evolutionary scenario of the vertebrate cerebellum, as shown in Figure 4. The developing brain of the last common ancestor of extant vertebrates already had *Otx-Hox* free r1, with isthmic organizer signals such as FGF8 and Wnt1. Given the presence of similar expression field with conserved function in the hemichordate larvae, the isthmic FGF signaling may date back to deep deuterostome ancestry (Pani et al., 2012). In the r1 rhombic lip, *Pax6* and *Wnt1* specify a domain, from which the granule cells differentiate as the result of *Atoh1* and *NeuroD* expression. Also, *Ptf1a*-positive ventricular zone generates some GABAergic inhibitory neurons.

We can also trace lineage-specific evolutionary modifications to this ancestral state of the cerebellum. In the hagfish, although the rhombic lip and ventricular zone are present in the embryo, most structures and neurons were degenerated developmentally in the adult brain.

Unlike in gnathostomes and hagfish, lamprey *Pax6a* and *6b* are not expressed in the rhombic lip (Murakami et al., 2001; Sugahara et al., 2016). In *Pax6* knockout mice, proliferation and initial differentiation of granule cells are unaffected, while cell migration to form the EGL is disrupted (Engelkamp et al., 1999). So far, migration of granule cells has not been observed in the lamprey as in cartilaginous fish and paddle fish. It is possible that the lamprey lost this *Pax6* expression domain in the rhombic lip secondarily. Subsequently, *Pax6* may have become involved in the migration of granule cells and the formation of the EGL at a certain period of gnathostome evolution (Figure 4). Recently, a third lamprey *Pax6* gene was identified (Ravi et al., 2019). Future experiments will be necessary to determine whether this *Pax6* paralog is expressed in the lamprey rhombic lip or not, which eventually could change this evolutionary scenario (Figure 4).

The cerebellar cortex was established in gnathostomes before the divergence of chondrichthyans and osteichthyans. At this period, Purkinje cells started expressing *Shh*, which is not seen in the lampreys (Sugahara et al., 2011). Finally, the proliferative activity of EGL cells by SHH signaling was acquired in the course of amniote evolution. In conclusion, we suggest that the *Atoh1/NeuroD* axis for granule cell fate and *Ptf1a* role in inhibitory neuronal specification are genetic programs involved in cerebellar development that were already established in the last common ancestor of living vertebrates. Subsequently, the developmental program of the cerebellum has evolved to adapt to complex environments especially in gnathostomes, presumably related to the acquisition of balancing organs such as paired appendages and inner ear with three semicircular canals.

## Data Availability Statement

The datasets presented in this study can be found in online repositories. The names of the repository/repositories and accession number(s) can be found in the article/Supplementary Material.

## Ethics Statement

The animal study was reviewed and approved by the Animal Experiment Committee of Hyogo College of Medicine.

## Author Contributions

FS, JP-A, SKurat, and YM conceived the project, designed the experiments, and wrote the manuscript. FS performed animal sampling and gene cloning and gene expression analyses. JP-A searched gene sequences from NGS databases. SKurak performed the gene phylogenetic analysis. All authors analyzed and discussed the data, and approved the final version of the manuscript.

## Conflict of Interest

The authors declare that the research was conducted in the absence of any commercial or financial relationships that could be construed as a potential conflict of interest.

## Publisher’s Note

All claims expressed in this article are solely those of the authors and do not necessarily represent those of their affiliated organizations, or those of the publisher, the editors and the reviewers. Any product that may be evaluated in this article, or claim that may be made by its manufacturer, is not guaranteed or endorsed by the publisher.

## Supplementary Material

Supplementary Figure 1 Expression of genes involved signaling pathways of the isthmic organizer. *Wnt1* **(A,B)** and *FGF8/17* **(C,D)** in hagfish stage 45 **(A,C)** and lamprey stage 26 **(B,D)**. Arrowheads mark the isthmic organizer region in the most caudal part of the midbrain. lv, lateral ventricle; 4v, fourth ventricle; ph, pharynx.

Supplementary Figure 2 Temporal expression of the *NeuorD* in the rhombomere 1. Dorsal views of the head region of the lamprey stages 25 **(A)**, 26 **(B)**, and 27 **(C)**. Arrowheads in **(B,C)** indicate the expression in the rhombomere 1. gV_1_, ophthalmic ganglion; gV_2,3_, maxillomandibular ganglion.

Supplementary Figure 3 Phylogenetic trees of *Wnt1* **(A)** and *Ptf1a* **(B)** genes. The trees were inferred with the maximum-likelihood method using 355 and 153 aligned amino acid sites, respectively. The support values at nodes indicate bootstrap values and posterior probabilities based on the maximum-likelihood method and Bayesian inference in order, respectively. See the section “Materials and Methods” for details.

Supplementary Table 1 Primers used for gene cloning and probe synthesis.
