# Supplementary figures and images for: Genetic Mechanism for the Cyclostome Cerebellar Neurons Reveals Early Evolution of the Vertebrate Cerebellum
Source: Front Cell Dev Biol. 2021 Aug 18;9:700860. doi: 10.3389/fcell.2021.700860 (PMC8416312; doi:10.3389/fcell.2021.700860)

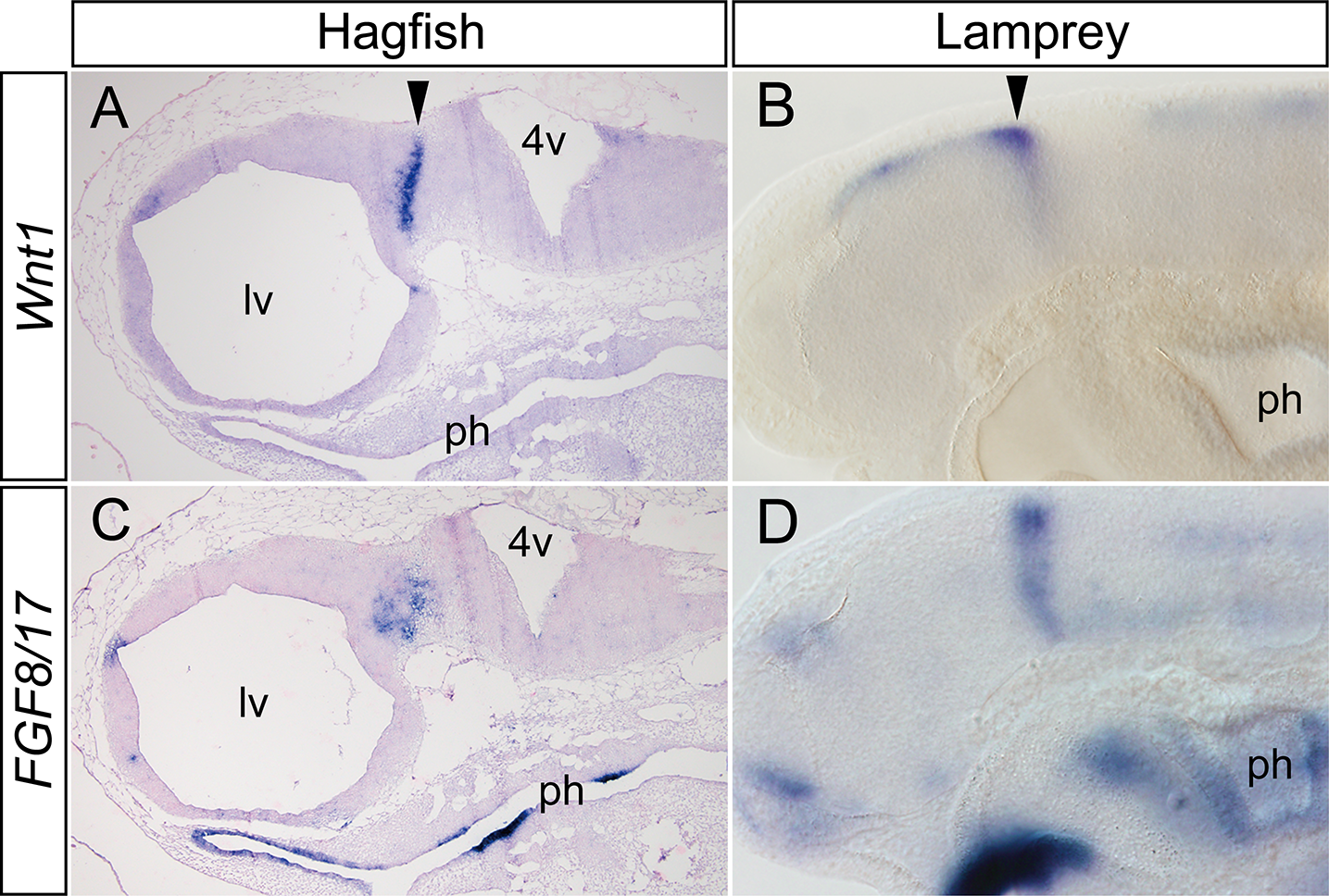

Supplement: Supplementary Figure 1 — Expression of genes involved signaling pathways of the isthmic organizer. Wnt1 (A,B) and FGF8/17 (C,D) in hagfish stage 45 (A,C) and lamprey stage 26 (B,D). Arrowheads mark the isthmic organizer region in the most caudal part of the midbrain. lv, lateral ventricle; 4v, fourth ventricle; ph, pharynx. [file Image_1.TIF]

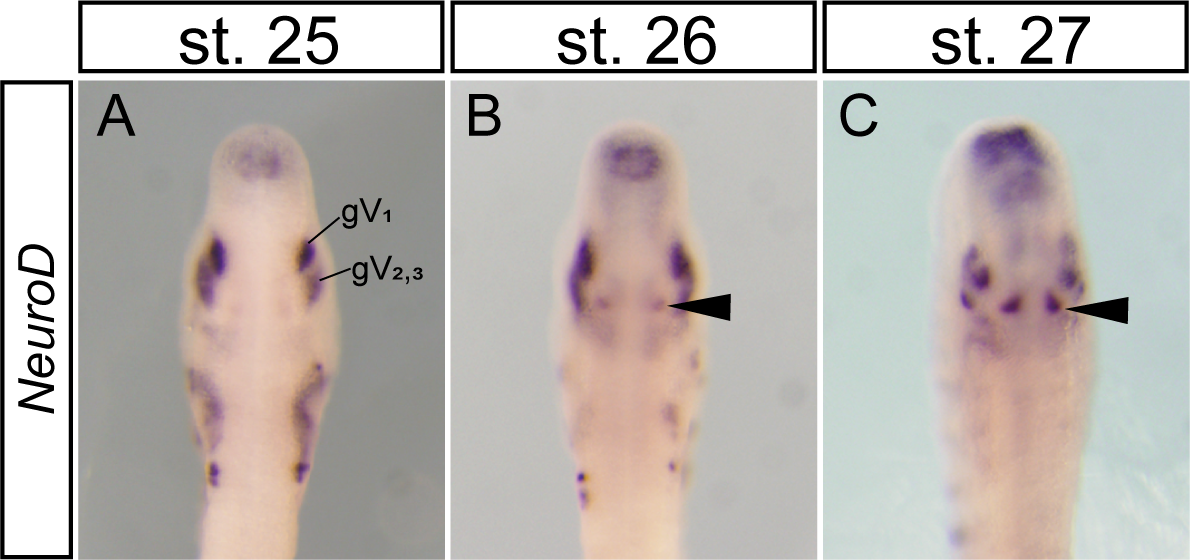

Supplement: Supplementary Figure 2 — Temporal expression of the NeuorD in the rhombomere 1. Dorsal views of the head region of the lamprey stages 25 (A), 26 (B), and 27 (C). Arrowheads in (B,C) indicate the expression in the rhombomere 1. gV1, ophthalmic ganglion; gV2,3, maxillomandibular ganglion. [file Image_2.TIF]

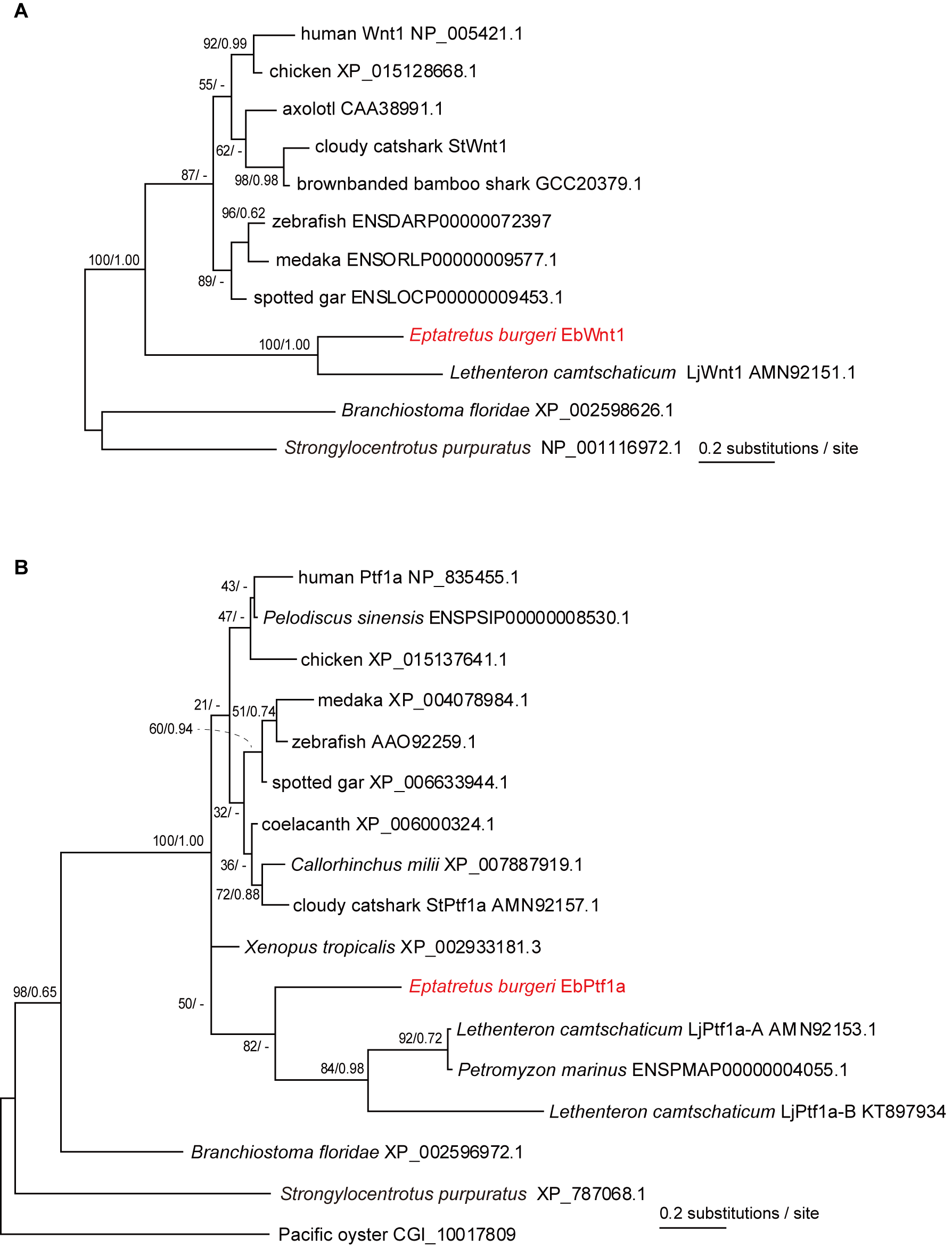

Supplement: Supplementary Figure 3 — Phylogenetic trees of Wnt1 (A) and Ptf1a (B) genes. The trees were inferred with the maximum-likelihood method using 355 and 153 aligned amino acid sites, respectively. The support values at nodes indicate bootstrap values and posterior probabilities based on the maximum-likelihood method and Bayesian inference in order, respectively. See the section “Materials and Methods” for details. [file Image_3.TIF]
