# Supplementary material for: Genetic Mechanism for the Cyclostome Cerebellar Neurons Reveals Early Evolution of the Vertebrate Cerebellum
Source: Front Cell Dev Biol. 2021 Aug 18;9:700860. doi: 10.3389/fcell.2021.700860 (PMC8416312; doi:10.3389/fcell.2021.700860)
Supplement: Supplementary Table 1 — Primers used for gene cloning and probe synthesis. [file Table_1.docx]

**Supplementary table 1.** Primers used for gene cloning and probe synthesis.

| **Species** | **Genes** | **Primer names** | **Sequences (5' to 3')** | **PCR** |
| --- | --- | --- | --- | --- |
| *E.burgeri* | *Ptf1a* | Ptf1a-F | GGCGTGAGTTCTACCAAAAGG | 1st PCR by F and R |
|  |  | Ptf1a-R | TTACAAGTGCCCCCCATTTTG | Nested PCR by nestedF and nestedR |
|  |  | Ptf1a-nestedF | GTGAGTTCTACCAAAAGGAGACG |  |
|  |  | Ptf1a-nestedR | TTCCTGAAAGATGATGGAGTCA |  |
| *E.burgeri* | *Wnt1* | Wnt1-F | ATGCTTTGCCTCTCTAATCGC | 1st PCR by F and R |
|  |  | Wnt1-R | TCACAAGCACTCGTGCACG | Nested PCR by nestedF and nested R |
|  |  | Wnt1-nestedF | CTTTGCCTCTCTAATCGCTTG | PCR for probe by probeF and nestedR |
|  |  | Wnt1-nestedR | CAAGCACTCGTGCACGGTC |  |
|  |  | Wnt1-probeF | GGAGATCGTCCACTCCGGC |  |
| *L.camtschaticum* | *GAD* | GAD-F | TGGCCTGGACATCATTGGATTG |  |
|  |  | GAD-R | GCTGAGCTTGGCTCTCCTCT |  |
